# Oropharyngeal Candidosis in HIV-Infected Patients—An Update

**DOI:** 10.3389/fmicb.2018.00980

**Published:** 2018-05-15

**Authors:** Shankargouda Patil, Barnali Majumdar, Sachin C. Sarode, Gargi S. Sarode, Kamran H. Awan

**Affiliations:** ^1^Division of Oral Pathology, Department of Maxillofacial Surgery and Diagnostic Sciences, College of Dentistry, Jazan University, Jizan, Saudi Arabia; ^2^Department of Oral Pathology and Microbiology, Bhojia Dental College & Hospital, Baddi, India; ^3^Department of Oral Pathology and Microbiology, Dr. D.Y. Patil Dental College and Hospital, Dr. D.Y. Patil Vidyapeeth, Pimpri, India; ^4^College of Dental Medicine, Roseman University of Health Sciences, South Jordan, UT, United States

**Keywords:** AIDS, antifungals, *Candida*, HIV, opportunistic infections, Oropharyngeal candidosis

## Abstract

Oropharyngeal candidosis (OPC) is an opportunistic fungal infection that is commonly found in HIV-infected patients, even in the twenty-first century. *Candida albicans* is the main pathogen, but other *Candida* species have been isolated. OPC usually presents months or years before other severe opportunistic infections and may indicate the presence or progression of HIV disease. The concept of OPC as a biofilm infection has changed our understanding of its pathobiology. Various anti-fungal agents (both topical and systemic) are available to treat OPC. However, anti-fungal resistance as a result of the long-term use of anti-fungal agents and recurrent oropharyngeal infection in AIDS patients require alternative anti-fungal therapies. In addition, both identifying the causative *Candida* species and conducting anti-fungal vulnerability testing can improve a clinician's ability to prescribe effective anti-fungal agents. The present review focuses on the current findings and therapeutic challenges for HIV-infected patients with OPC.

## Introduction

Oropharyngeal candidosis (OPC) is the most prevalent and recurrent opportunistic infection in acquired immune deficiency syndrome (AIDS) patients and often indicates the presence of human immunodeficiency virus (HIV) infection and its progression (Li et al., 2012). According to Flint et al. (2006), OPC meets the criteria to serve as a useful marker for both the restoration of immune functions and HIV disease progression following highly active antiretroviral therapy (HAART) failure, because it is not gender- or race-specific, it occurs early in immune dysfunction in the erythematous form, and its prevalence correlates with the HIV viral load. Although the occurrence of OPC has declined following the introduction of anti-retroviral therapy (ART), it remains a substantial problem for patients in resource-limited locales or among individuals who develop mycological resistance or have a poor immunologic response (Thompson et al., 2010).

OPC denotes candidosis of the mouth and throat; when the disease involves the esophagus, it is termed esophageal candidosis or candida esophagitis (Centers for Disease Control and Prevention, 2017). OPC inevitably occurs in ~80–90% of HIV patients in the primary, asymptomatic, or overt phases of the disease (Vazquez, 2000). It primarily manifests as a superficial mucosal infection in the form of pseudomembranous, erythematous, or angular cheilitis in HIV-positive individuals. The chronic atrophic candidosis (denture stomatitis) variant is often seen in the aging HIV-infected population due to their improved survival. Manifestations in the form of kissing lesions (concomitant lesions on the tongue and palate) can also be pathognomonic presentations of HIV-associated candidiasis. Patients complain chiefly of pain and a burning sensation in the mouth and an altered (cotton-like) taste sensation. Extensive involvement of the esophagus often leads to pain and difficulty in swallowing, eventually resulting in esophageal candidosis (Thompson et al., 2010; Cassone and Cauda, 2012).

The detailed pathogenesis underlying the predisposition of HIV-infected patients for OPC is unclear. Evidence provided by several studies indicates that pathogenic *Candida* colonization in an uninfected individual is shielded by a concerted contribution of oral keratinocytes, immune cells, and salivary constituents; thus, multiple rather than single defects in the host defense mechanism seem to increase the risk of OPC in HIV patients (Repentigny et al., 2004). Moreover, *C. albicans* is hypothesized to form complex biofilms on both mucosal and abiotic surfaces, such as dentures, and can efficaciously co-exist with oral commensal bacteria and host cells (Dongari-Bagtzoglou et al., 2009; Harriott and Noverr, 2011). *Candida*-bacterial biofilms have been proposed to have an increased likelihood of resistance to routine anti-fungal agents and are challenging to treat, especially in patients with oral prosthetic appliances (Jenkinson and Douglas, 2002; Nett et al., 2010).

HAART regimens have changed the prognosis of AIDS from a fatal condition to a chronic disease with a decent life expectancy. The use of HAART improves immunological functions and suppresses the viral load, thereby aiding in the decline of the majority of opportunistic infections, including OPC (Vazquez, 2010; Tamí-Maury et al., 2011). Nonetheless, the epidemiology of OPC in the present era of ART is not well-established. Furthermore, an epidemiological shift of the common pathogen *C. albicans* to resistant *C. albicans* and the rise in intrinsically resistant non-*C. albicans Candida* (NCAC) species has been acknowledged despite the judicious use of fluconazole, which is the chief azole anti-fungal drug used to treat primary OPC cases in HIV-infected patients (Patel et al., 2012; Berberi et al., 2015).

Thus, with the above outlook, the present narrative review emphasizes the changing *Candida* profiles and the subsequent challenges in the diagnosis and management of OPC in HIV-positive patients.

### Method

A web-based search was performed via the PubMed database with the keywords OPC, epidemiology, prevalence, HIV/AIDS, *Candida* biofilm, anti-fungal resistance, diagnosis, and management. Original research (2007–2017, studies involving epidemiological data stating OPC, patients on/off ART and anti-fungal with/without symptomatic OPC), reviews, case reports and short communications published in the English language were included to appraise various topics. The findings are noted in the following sub-sections, followed by a discussion under the current therapeutic challenges section.

## *Candida* profile in aids patients in the post-haart era

### Epidemiology of OPC over the past decade

The selected studies (Table 1) revealed that the prevalence of OPC ranged from 0.9 to 83% (Adedigba et al., 2008; Nadagir et al., 2008; Fabian et al., 2009; Thompson et al., 2010; Tamí-Maury et al., 2011; Patel et al., 2012; Kwamin et al., 2013; Maurya et al., 2013; Mulu et al., 2013; Berberi et al., 2015; Kirti et al., 2015; Das et al., 2016; Konaté et al., 2017; Terças et al., 2017). The reported studies from African populations showed that the prevalence of OPC ranged from 0.9 to 81.5% (Adedigba et al., 2008; Fabian et al., 2009; Kwamin et al., 2013; Mulu et al., 2013; Konaté et al., 2017). The studies from the Indian sub-continent showed that the prevalence of OPC ranged from 5.0 to 38.8% (Nadagir et al., 2008; Maurya et al., 2013; Kirti et al., 2015; Das et al., 2016).

**Table 1:** Occurrence of OPC in AIDS patients.

**S. No**.	**Author, Year**	**Region**	**Sample size**	**OPC cases (%)**	**On ART**	**On Anti-fungal**
1.	Terças et al., 2017	Brazil	52	43 (83%)	Few	Few (52.2%)
2.	Konaté et al., 2017	Cote d'Ivoire	286 (281 HIV+)	227 (79.4%)	88.5%	No
3.	Das et al., 2016	India	141	27 (19.1%)	No	Few (17/141)
4.	Berberi et al., 2015	Lebanon	50	38 (76%)	Yes	Not mentioned
5.	Kirti et al., 2015	India	100	20 (20%)	75/100	Not mentioned
6.	Kwamin et al., 2013	Ghana	267	66 (81.5%) 147 (79%)	Yes (81/267) No (186/267)	No
7.	Mulu et al., 2013	Northwest Ethiopia	221	82 (37.5%)	Yes	Few
8.	Maurya et al., 2013	India	190	16/90 (17.8%) 5/100 (5.0%)	No Yes	No
9.	Patel et al., 2012	Texas	215	59 (27%)	30/59	Few (79/215)
10.	Tamí-Maury et al., 2011	Alabama	375	281 (74.9%)	Few	Not mentioned
11.	Thompson et al., 2010	Texas	122	99 (81.1%) (33.3% symptomatic)	Yes	Yes
12.	Fabian et al., 2009	Tanzania	187	12 (6.4%)	No	Not mentioned
13.	Nadagir et al., 2008	India	340	132 (38.8%)	Yes	Yes
14.	Adedigba et al., 2008	Nigeria	225	2 (0.9%)	No	Not mentioned

### Prevalent *Candida* species

The collected data from the relevant studies (Table 2) conducted over the past decade depicted *C. albicans* as the most prevalent *Candida* species (37.2–95.2%) in HIV patients with OPC. *C. glabrata, C. tropicalis, C. parapsilosis, C. krusei*, and *C. dubliniensis* were found in decreasing order among the NCAC species. *C. glabrata* (0.99–23.0%) and *C. tropicalis* (1.2–12%) were invariably found with *C. albicans* in most studies (Nadagir et al., 2008; Thompson et al., 2010; Patel et al., 2012; Kwamin et al., 2013; Maurya et al., 2013; Mulu et al., 2013; Sharifzadeh et al., 2013; Ho et al., 2014; Berberi et al., 2015; Katiraee et al., 2015; Menezes et al., 2015; Das et al., 2016; Konaté et al., 2017; Terças et al., 2017). *C. dubliniensis* was found relatively more often in two of the Indian studies and two studies from Texas (Nadagir et al., 2008; Thompson et al., 2010; Patel et al., 2012; Das et al., 2016).

**Table 2:** Isolation and frequency of different *Candida* species with respect to OPC in HIV-positive patients.

**S. No**.	***Candida* species**		**Frequency of isolates (%)**												
1.	*C. albicans*	56	95.2	77.0	67.6	92.0	60	74.0	68.50	90.5	37.2	46.0	62	54	66.6
2.	*C. glabrata*	8	1.3	3.2	4.5	2.6	23	6.5	0.99	4.8	19.4	5.6	17	16	–
3.	*C. tropicalis*	12	2.2	–	7.2	5.3	5	4.5	7.39	1.2	6.9	7.0	5	6	8.9
4.	*C. parapsilosis*	4	0.4	3.2	9.0	–	3	1.9	2.96	–	6.5	–	1	2	11
5.	*C. krusei*	12	–	–	3.6	–	2	–	6.40	1.1	7.3	0.009	2	3	20
6.	*C. dubliniensis*	–	–	14.7	2.7	–	5	6.5	1.48	–	7.7	–	12	17	48.9
7.	*C. kefyr*	–	–	–	1.8	–	2	–	0.49	2.4	–	–	–	–	–
8.	*C. famata*	4	–	1.6	0.9	–	–	0.6	0.99	–	–	–	–	–	–
9.	*C. guilliermondii*	4	–	–	0.9	–	–	1.9	0.99	–	2.0	–	–	0.5	4.9
10.	*C. lusitaniae*	–	–	–	0.9	–	–	–	0.99	–	2.0	–	–	1	–
11.	*C. sake*	–	–	–	–	–	–	–	2.46	–	–	–	–	–	–
12.	*C. stellatoidea*	–	–	–	–	–	–	–	–	–	–	–	–	–	6.7
**Author, Year**		Terças et al., 2017	Konaté et al., 2017	Das et al., 2016	Menezes et al., 2015	Berberi et al., 2015	Katiraee et al., 2015	Ho et al., 2014	Kwamin et al., 2013	Maurya et al., 2013	Sharifzadeh et al., 2013	Mulu et al., 2013	Patel et al., 2012	Thompson et al., 2010	Nadagir et al., 2008

### Mixed *Candida* colonization

Mixed colonization by various NCAC species together with *C. albicans* has been reported to occur in 23.7% of cases (Mulu et al., 2013). Ho et al. (2014) reported that among the 45% of HIV outpatients colonized by yeasts, 16.5% harbored more than one species. Similarly, Menezes et al. (2015) reported that 77.5% of the cases had *Candida* colonization by a single species, whereas 22.5% of the cases had a combination of two or more species. In another study, 7 of the 43 considered patients had double colonization (Terças et al., 2017).

### Anti-fungal-resistant *Candida* species

In several of the studies, the frequency of fluconazole-resistant *C. albicans* was reported to be 9.3, 12.2, 16, 17.6, 25.97, and 56.7%, whereas the frequency of fluconazole-resistant *C. glabrata* was reported to range from 50 to 52% (Nadagir et al., 2008; Mulu et al., 2013; Ho et al., 2014; Katiraee et al., 2015; Rosana et al., 2015; Salari et al., 2016; Terças et al., 2017). *C. glabrata* possesses the ability to develop resistance to fluconazole after exposure, whereas *C. krusei* has innate resistance to this anti-fungal agent (Terças et al., 2017). Similarly, *C. lusitaniae* exhibits a unique tendency to readily develop resistance to anti-fungal agents, such as fluconazole, amphotericin B, and flucytosine (Zhang et al., 2012).

### *Candida* colonization and CD4+ cell counts

Several studies have shown a significant correlation between CD4+ T lymphocytes and *Candida* colonization. A lower count, especially below 200 CD4+ cells with or without statistical significance has been frequently associated with the increased occurrence of OPC (Tamí-Maury et al., 2011; Maurya et al., 2013; Ho et al., 2014; Berberi et al., 2015; Kirti et al., 2015; Menezes et al., 2015; Das et al., 2016; Konaté et al., 2017). The Th17 cell functional subset within the CD4+ T cell lineage seems to be selectively depleted with the progression of HIV infection and appears to be the critical host determinant of the ability of *C. albicans* to overwhelm epithelial defenses and cause disease (Cassone and Cauda, 2012).

### *Candida* colonization and viral load

The data on the association between the plasma HIV RNA level and OPC differ and are not conclusive (Li et al., 2012). Only a few recently conducted studies have shown a correlation between high viral loads and an increased frequency of OPC in HIV-infected patients (Tamí-Maury et al., 2011; Ho et al., 2014; Terças et al., 2017).

### *Candida* biofilm

Complex oral *Candida* biofilms have been reported to be comprised of fungal, bacterial and host cells or cell-derived products (Dongari-Bagtzoglou et al., 2009). An *in vivo* study in rats demonstrated that the *Candida* biofilms associated with dentures were more resistant to common anti-fungal drugs and showed a relatively mixed-species colonization (Nett et al., 2010). The interactions between one common commensal of the oral cavity (*Streptococcus gordonii*) and *C. albicans* has been suggested to influence the development of biofilms via physical (adherence) and chemical (diffusible) signals (Harriott and Noverr, 2011).

## Current therapeutic challenges

The interpretations of the above findings from recent studies on OPC in HIV patients are as follows:

### Art impacts the occurrence of OPC in AIDS patient, but OPC is still prevalent in the present era in a varying range (0.9–83%)

Protease inhibitors (PI) were the earliest anti-retrovirals proposed to be linked to a decreased rate of OPC in AIDS patients (Ho et al., 2014). The HIV proteinase inhibitors in the PI-HAART cocktail impede *Candida* SAP (HIV proteinases share an elevated sequence homology with *C. albicans* SAP), resulting in an initial dramatic reduction in OPC (Cassone and Cauda, 2012). The reduction of OPC following PI-HAART is also attributed to immune reconstitution, as measured by the elevation of circulating CD4+ T cells (CD4) and the reduction of the viral load (Tamí-Maury et al., 2011).

The studies quoted in Table 1 state the prevalence of OPC, but very few of these studies mention clear inclusion and exclusion criteria, which are essential for true determination of the impact of ART on OPC. The studies conducted by Konate et al., Kwamin et al., and Maurya et al. clearly defined the selected HIV-positive patients and their anti-fungal and ART statuses. The findings of Konaté et al. (2017) revealed a high prevalence of OPC (79.4%) despite the patients undergoing ART. These authors suggested this finding might have occurred because PIs were not used as a first line of ART in Côte d'Ivoire, the majority of the patients had CD4+ cell counts below 200 and a high proportion of the patients were under concurrent tuberculosis treatment regimens with rifampicin. Kwamin et al. and Maurya et al. divided the HIV-positive patients into two groups (i. e., the first under HAART and the second without a HAART regimen). The difference in the prevalence of OPC among the two groups was insignificant in the study of Kwamin et al. (2013). Conversely, Maurya et al. (2013) reported an interesting finding; although a significant difference was noted in the OPC occurrence, ART did not affect *Candida* colonization between the two groups. Similarly, Thompson et al. (2010) observed high yeast colonization (81.1%) and low symptomatic OPC infection (33.3%) in HIV-positive patients undergoing ART and anti-fungal treatment. Thus, ART appears to decrease the symptomatic manifestation of OPC rather than *Candida* colonization.

Another explanation for the prevalence of OPC may be HIV-IRIS (HIV-associated immune reconstitution inflammatory syndrome), which usually manifests within the first 6 months of ART initiation. This disorder refers to a pathological inflammatory response that is typically directed toward microbial antigens due to immune recovery following the commencement of ART. Two distinct patterns of the disorders have been recognized: “paradoxical IRIS” (wherein the signs and symptoms of a diagnosed opportunistic infection worsen acutely despite receiving treatment with a favorable response) and “unmasking IRIS” (wherein a new opportunistic infection with a pronounced inflammatory component develops). OPC is suggested to be a likely manifestation of the unmasking type of HIV-IRIS (Walker et al., 2015). Nonetheless, limited studies have investigated the oral opportunistic lesions associated with HIV-IRIS. Ramírez-Amador et al. (2009) and Gaitan Cepeda et al. (2008) reported that OPC was a distinct manifestation of this syndrome. Achenbach et al. (2011) reported that *Candida* esophagitis was a consequence of paradoxical IRIS.

### Although *C. albicans* is the most common opportunistic pathogen, NCAC species, especially *C. glabrata* and *C. tropicalis*, together with mixed species colonization are significantly reported

*C. glabrata* is the most common NCAC species isolated from HIV-positive patients, as evidenced from Table 2. Exposure to anti-fungal agents during treatment of candidosis may provide positive selection pressure for NCAC species, such as *C. glabrata* and *C. krusei*, which are considered intrinsically less sensitive than other species to anti-fungal agents, thereby increasing their prevalence (Pfaller, 2012). Furthermore, mixed *C. albicans* and *C. glabrata* co-infections are more challenging to treat. Alves et al. (2014) investigated *in vitro* co-infection by *C. albicans* and *C. glabrata* in a reconstituted human vaginal epithelium and observed higher tissue damage in the co-infection compared to the single *C. albicans* infection. Recently, Tati et al. (2016) demonstrated that *C. albicans* aided in both the initial colonization and establishment of OPC infection by *C. glabrata*, which was suggestive of a synergistic relationship. Interactions between *C. albicans* and other NCAC species have been investigated, and *C. dubliniensis* and *C. krusei* have been observed to suppress *C. albicans* populations in biofilms. However, both *in vivo* and *in vitro* study models have shown that single infections by *C. albicans* are more harmful than mixed infections with *C. albicans—C. glabrata* or *C. albicans—C. krusei* (Rossoni et al., 2015).

### Resistant *Candida* species are frequently associated with AIDS

Acquired resistance is uncommon but has been reported to be escalating at present (Cassone and Cauda, 2012; Pfaller, 2012; Sanguinetti et al., 2015). The collected data show that the frequency of resistant *C. albicans* in HIV-positive patients ranges from 9.3 to 56.7% (Nadagir et al., 2008; Mulu et al., 2013; Ho et al., 2014; Katiraee et al., 2015; Rosana et al., 2015; Salari et al., 2016; Terças et al., 2017). Acquired resistance to fluconazole is mainly attributed to repeated and prolonged exposure or suppressive courses of low doses of the drug combined with severe immune suppression (Patton et al., 2001; Lortholary et al., 2012; Liu et al., 2015). Patient compliance is another vital factor that may shroud the true reflection of a drug's efficacy (Patton et al., 2001). Furthermore, to adapt to various host niches under stressful conditions, *C. albicans* regulates gene expression and biochemical activities according to cellular needs by engendering genetically altered variants as an adaptive response to the changing host environment and new niches, thereby endowing it with anti-fungal drug resistance (Hampe et al., 2017).

Cross-resistance refers to non-susceptibility to several drugs of the same class. For example, specific FKS1 mutations in *C. albicans* produce cross-resistance to all echinocandins. Multi-drug resistance refers to simultaneous resistance to at least two different classes of anti-fungal agents. Loss-of-function mutations in ERG3 in *C. albicans* and *C. dubliniensis* have been reported to result in simultaneous multi-drug resistance to azoles and amphotericin B (Sanglard, 2016). Mulu et al. (2013) reported cross-resistance in 9 *Candida* isolates from OPC patients with AIDS that were resistant to fluconazole and concurrently were found to be resistant to ketoconazole (2 isolates) and itraconazole (5 isolates). The combined overexpression of CDR2 and ERG11 and a mutation in the ERG11 gene were found to be a genetic mechanism of fluconazole resistance in *C. albicans* isolated from HIV patients in Indonesia (Rosana et al., 2015). Brief descriptions of the resistance mechanisms of important classes of anti-fungal agents have been compiled in Table 3 (Kanafani and Perfect, 2008; Pfaller, 2012; Vandeputte et al., 2012; Morace et al., 2014; Patil et al., 2015; Sanguinetti et al., 2015; Sanglard, 2016; Hampe et al., 2017).

**Table 3:** Major classes of anti-fungal drugs and mechanism of resistance in *Candida* species (Kanafani and Perfect, 2008; Pfaller, 2012; Vandeputte et al., 2012; Morace et al., 2014; Patil et al., 2015; Sanguinetti et al., 2015; Sanglard, 2016; Hampe et al., 2017).

**Anti-fungal**	**Mechanism of action**	**Mechanism of resistance development**
Azoles	Inhibit the target enzyme lanosterol 14-a-sterol demethylase, which aids in the conversion of lanosterol to ergosterol (an important component of the fungal cell membrane), resulting in accumulation of the toxic product 14-a-methyl-3,6-diol	Development of active efflux pumps (facilitated by up regulation of the CDR1, CDR2 and MDR1 genes) Prevents binding to the target enzyme lanosterol C14a-demethylase site (mutations in the ERG11 gene) Target enzyme up-regulation (higher intracellular ERG11p concentrations Prevents the formation of 14a-methyl-3,6-diol (a toxic product) from 14a-methylfecosterol and enables functional membranes (mutation of the ERG3 gene) Gain of function mutations in Mrr1, Tac1 and Upc2 (zinc cluster transcription factors)
Polyenes	Formation of porin channels leading to loss of transmembrane potential and impaired cellular function	Defects in the ERG3 gene Increased catalase activity
Echinocandins	Inhibit the synthesis of b-1,3-D glucan, which is an integral component of the fungal cell wall	Point mutations in the Fks1 gene Increase in chitin synthesis in *Candida* species Paradoxical effect
Pyrimidine analog	Inhibits cellular DNA and RNA synthesis	Mutation in cytosine permease Defects in flucytosine metabolism through mutations in cytosine deaminase or uracil phosphoribosyl transferase (FUR1 gene mutations)

### Candida biofilms are more resistant to treatment

The key aspect of *C. albicans* pathobiology is formation of a biofilm, which is regulated by six transcription factors (Bcr1, Brg1, Efg1, Ndt80, Rob1, and Tec1; Glazier et al., 2017). *Candida* is also known to form mixed-bacteria or polymicrobial biofilms that are capable of surviving many external challenges; for example, mixed biofilms containing *C. albicans* and *Streptococcus* spp. are more resistant to anti-microbial treatments (Thein et al., 2009; Barros et al., 2016). Pertaining to the oral cavity, the chief example of this type of association is *Candida*-associated denture stomatitis, wherein *Candida* coexists with several bacterial species, such as *S. aureus, E. coli*, and *Klebsiella* species (Thein et al., 2009). Thus, biofilms are difficult to eradicate and may cause frequent relapses and recurrent cases of OPC, especially in immunocompromised patients, such as AIDS patients. Several techniques have been advocated for the inhibition of biofilm formation, such as the use of silver nanoparticles, monoclonal antibodies, photodynamic therapy, enzymatic degradation of biofilm components, and the use of probiotic organisms to alter the structure, physiology, and behavior of mixed-species biofilms (Thein et al., 2009; Sardi et al., 2014).

### Various drug interactions may play a role in the success of anti-fungal treatment

The coexistence of epidemics of HIV, tuberculosis and malaria and opportunistic infections, such as those found in Africa, make drug interactions unavoidable to a great extent (Kigen et al., 2011). In one study, interactions involving nevirapine and ketoconazole revealed a 72% decrease in the ketoconazole concentration upon co-administration. The concurrent administration of fluconazole and nevirapine resulted in a 33% increase in nevirapine and thus was a probable cause of toxicity (Kigen et al., 2011).

Most tuberculosis regimens include rifampicin, which is a potent inducer of cytochrome P450 enzymes. The concomitant administration of rifampicin with fluconazole has led to noteworthy changes in the pharmacokinetic parameters of fluconazole, including a 39% increase in its elimination rate constant and a 28% shorter elimination half-life in AIDS patients (Panomvana Na Ayudhya et al., 2004). Similarly, the concurrent administration of rifampicin with ketoconazole and itraconazole markedly reduces the serum concentrations of these anti-fungal drugs (Swart and Harris, 2005). In one study, two patients co-infected with HIV-tuberculosis on both anti-tubercular drugs and azoles were reported to present for several months with recurrent episodes of OPC despite the presence of susceptible *Candida* strains, which could be explained by the previously discussed drug interactions (Mulu et al., 2013).

The occurrence of OPC in HIV-infected patients is determined by a multitude of factors, including the immune status and constitution of the individual, host cell-bacteria-mycological interactions, patient compliance, the response to anti-fungal therapy, anti-viral drug interactions, immune reconstitution, accessibility to standard treatment, and the severity of the immunocompromised status. Thus, the challenge of controlling and treating OPC in HIV patients can be met by identifying the *Candida* species and appropriate choice of the anti-fungal agent in combination with ART. Furthermore, the virulence factors and biofilm formation of the *Candida* organisms may be negated with exploration of the genetic pathways.

## Diagnosis

**Figure 1:**
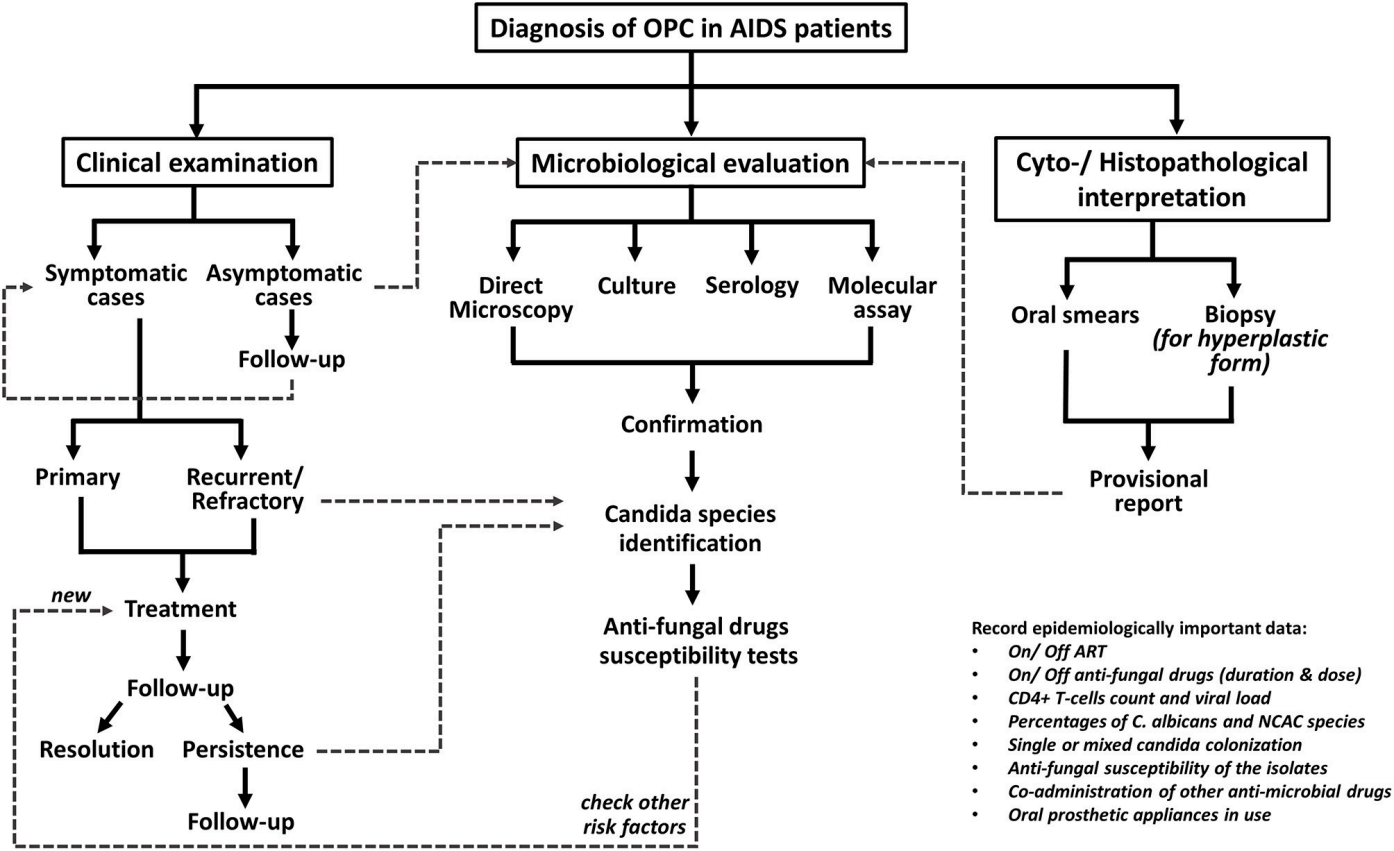
Diagnostic protocol for oropharyngeal candidiasis in HIV-positive patients.

The diagnosis of OPC is fairly clinical and is based on presentation with classical signs and symptoms of candidosis, although advanced microbiological assays are obligatory in non-responding cases. The diagnostic procedure for OPC cases in at-risk HIV patients is depicted in Figure 1 (Thompson et al., 2010; Lortholary et al., 2012; Patil et al., 2015; Kaur et al., 2016).

## Management

Currently, several anti-fungal agents are available for OPC treatment (Table 4). Topical therapies are implicated for milder presentations of OPC. Generally, fluconazole is the leading drug of choice and is notably effective with appropriate compliance with the HAART regimen (Vazquez, 2010; Lortholary et al., 2012; Patil et al., 2015). Among the other azoles, posaconazole is a superior option for therapy in cases with fluconazole-resistant *Candida* species (Vazquez, 2010; Katragkou et al., 2012). Itraconazole usage is complicated by its cross-resistance to fluconazole (Lortholary et al., 2012). The administration of ketoconazole is limited due to its adverse side effects and drug interactions with some HIV protease inhibitors (Vazquez, 2010). Echinocandins are effective due to their unique mechanisms of action and lack overlap resistance with the triazoles; thus, they are beneficial in refractory cases (Patil et al., 2015). Furthermore, although fluconazole is effective, anti-fungal prophylaxis for the prevention of OPC and esophageal candidiasis with this drug is not recommended, because very low attributable morbidity and mortality are associated with OPC. Additionally, fluconazole may lead to the development of drug-resistant *Candida* strains, although further acute therapy is highly effective and can cause significant drug-drug interactions ((Kanafani and Perfect, 2008; Lortholary et al., 2012)).

**Table 4:** Treatment of OPC (Vazquez, 2010; Lortholary et al., 2012; Patil et al., 2015).

**Anti–fungal agents**		**Form**	**Dosage**
First line	Nystatin Clotrimazole Miconazole Fluconazole	Suspension (100,000 U/mL) Pastilles (200,000 U each) Oral troche (10 mg) Lauriad Muco-adhesive buccal Tablets (50 mg) Tablet (100 mg−200 mg) Solution (10 mg/mL) (PO or I.V.)	4–6 mL four times daily for 7–14 days 1–2 mL four times daily for 7–14 days 1 five times daily for 7–14 days Once daily for 7–14 days Once daily for 7–14 days (Apply to mucosal surface following detailed manufacturer instructions) 1 tablet daily for 7–14 days 10 mL once daily for 7–14 days
Second line	Itraconazole Posaconazole Voriconazole	Capsule (200 mg) (PO) Solution (10 mg/mL) (PO) Tablet (400 mg) (PO) Tablet (200 mg) (PO or I.V.)	1 capsule daily for 28 days 10–20 mL once daily Daily in divided doses 2 tablets daily
Refractory cases	Caspofungin Micafungin Anidulafungin Amphotericin B Amphotericin B deoxycholate	I.V. I.V. I.V. Oral suspension I.V.	70 mg (loading dose) followed by 50 mg daily 100–150 mg daily 100 mg (loading dose) followed by 50 mg daily 500 mg every 6 h. 0.3 mg/kg once

Various alternative therapies have been suggested and investigated recently (Mehra et al., 2012). One such discovery includes chiloscyphenol A (extracted from Chinese liverworts), which shows promising fungicidal activity by inducing mitochondrial dysfunction and plasma membrane destruction in *C. albicans*. Furthermore, this drug exhibited potent activity in preventing biofilm formation by *C. albicans* and targeting cells within mature biofilms, including strains resistant to fluconazole (Zheng et al., 2018). Another study showed that all clinical *C. albicans* isolates were susceptible to the *Trachyspermum ammi* essential oil, which had a significant effect on fungal growth in the exponential phase (Sharifzadeh et al., 2015). Certain traditional Chinese medicinal herbs (crude extracts containing berberine, palmatine, allicin, pseudolaric acid A and B, magnolol, honokiol, and galangin) are known for their anti-*Candida* properties and are claimed to be decent choices for treating refractory OPC in AIDS patients (Liu et al., 2015). Other proposals recommended to counteract resistant *Candida* species include targeting of cationic peptides and various fungal virulence factors (Sharifzadeh et al., 2015).

## Conclusion

To summarize, OPC remains fairly prevalent in HIV-infected patients in the present era, with a significant percentage of NCAC and resistant *Candida* species being reported. Currently, fluconazole remains the leading drug of choice. The therapeutic implications of genetic pathways in *Candida* biofilm formation have not been explored. With the development of molecular and nano-technologies, the mechanisms of drug resistance are acknowledged more precisely and have vast implications for the development of targeted drug therapies.

## Author contributions

SP and KA: Conceptualized the paper; BM, SS, and GS: Are involved in literature search. All the authors have equally contributed in manuscript preparation, manuscript review, and manuscript editing.

### Conflict of interest statement

The authors declare that the research was conducted in the absence of any commercial or financial relationships that could be construed as a potential conflict of interest.

## References

[B1] Achenbach C. J. Harrington R. D. Dhanireddy S. Crane H. M. Casper C. Kitahata M. M. (2011). Paradoxical immune reconstitution inflammatory syndrome in HIV-infected patients treated with combination antiretroviral therapy after AIDS-defining opportunistic infection. Clin. Infect. Dis. 54, 424–433. 10.1093/cid/cir802 22095568 PMC3258272

[B2] Adedigba M. A. Ogunbodede E. O. Jeboda S. O. Naidoo S. (2008). Patterns of oral manifestation of HIV/AIDS among 225 Nigerian patients. Oral Dis. 14, 341–346. 10.1111/j.1601-0825.2007.01384.x 18410577

[B3] Alves C. T. Wei X. Q. Silva S. Azeredo J. Henriques M. Williams D. W. (2014). *Candida albicans* promotes invasion and colonisation of *Candida glabrata* in a reconstituted human vaginal epithelium. J. Infect. 69, 396–407. 10.1016/j.jinf.2014.06.002 24924556

[B4] Panomvana Na Ayudhya D. Thanompuangseree N. Tansuphaswadikul S. (2004). Effect of rifampicin on the pharmacokinetics of fluconazole in patients with AIDS. Clin. Pharmacokinet. 43, 725–732. 10.2165/00003088-200443110-00003 15301576

[B5] Barros P. P. Ribeiro F. C. Rossoni R. D. Junqueira J. C. Jorge A. O. C. (2016). Influence of Candida krusei and *Candida glabrata* on *Candida albicans* gene expression in *in vitro* biofilms. Arch. Oral Biol. 64, 92–101. 10.1016/j.archoralbio.2016.01.005 26803674

[B6] Berberi A. Noujeim Z. Aoun G. (2015). Epidemiology of oropharyngeal candidiasis in human immunodeficiency virus/acquired immune deficiency syndrome patients and CD4+ counts. J. Int. Oral Health 7, 20–23. 25878473 PMC4385720

[B7] Cassone A. Cauda R. (2012). Candida and candidiasis in HIV-infected patients: where commensalism, opportunistic behavior and frank pathogenicity lose their borders. AIDS 26, 1457–1472. 10.1097/QAD.0b013e3283536ba8 22472853

[B8] Das P. Saikia L. Nath R. Phukan S. K. (2016). Species distribution & antifungal susceptibility pattern of oropharyngeal candida isolates from human immunodeficiency virus infected individuals. Indian J. Med. Res. 143, 495–501. 10.4103/0971-5916.184288 27377507 PMC4928557

[B9] Centers for Disease Control Prevention (2017). USA: CDC; c2017. Candida Infections of the Mouth, Throat, and Esophagus; [about 1 screen]. Available online at: https://www.cdc.gov/fungal/diseases/candidiasis/thrush/index.html (Accessed January 11, 2018).

[B10] Dongari-Bagtzoglou A. Kashleva H. Dwivedi P. Diaz P. Vasilakos J. (2009). Characterization of mucosal *Candida albicans* biofilms. PLoS ONE 4:e7967. 10.1371/journal.pone.0007967 19956771 PMC2776351

[B11] Fabian F. M. Kahabuka F. K. Petersen P. E. Shubi F. M. Jurgensen N. (2009). Oral manifestations among people living with HIV/AIDS in Tanzania. Int. Dent. J. 59, 187–191. 10.1922/IDJ_2058Fabian05 19774801

[B12] Flint S. R. Tappuni A. Leigh J. Schmidt-Westhausen A. M. MacPhail L. (2006). (B3) markers of immunodeficiency and mechanisms of HAART therapy on oral lesions. Adv. Dent. Res. 19, 146–151. 10.1177/154407370601900126 16672565

[B13] Gaitan Cepeda L. A. Salobreña A. C. López Ortega K. Arzate Mora N. Jiménez Soriano Y. (2008). Oral lesions and immune reconstitution syndrome in HIV+/AIDS patients receiving highly active antiretroviral therapy. Epidemiological evidence. Med. Oral Patol. Oral Cir. Bucal. 13, E85–E93. Available online at: www.medicinaoral.com/pubmed/medoralv13_i2_p85.pdf 18223535

[B14] Glazier V. E. Murante T. Murante D. Koselny K. Liu Y. Kim D.. (2017). Genetic analysis of the *Candida albicans* biofilm transcription factor network using simple and complex haploinsufficiency. PLoS Genet. 13:e1006948. 10.1371/journal.pgen.1006948 28793308 PMC5565191

[B15] Hampe I. A. I. Friedman J. Edgerton M. Morschhäuser J. (2017). An acquired mechanism of antifungal drug resistance simultaneously enables *Candida albicans* to escape from intrinsic host defenses. PLoS Pathog. 13:e1006655. 10.1371/journal.ppat.1006655 28953977 PMC5633205

[B16] Harriott M. M. Noverr M. C. (2011). Importance of candida–bacterial polymicrobial biofilms in disease. Trends Microbiol. 19, 557–563. 10.1016/j.tim.2011.07.004 21855346 PMC3205277

[B17] Ho M. W. Yang Y. L. Lin C. C. Chi C. Y. Chen H. T. Lin P. C.. (2014). Yeast oropharyngeal colonization in human immunodeficiency virus-infected patients in Central Taiwan. Mycopathologia 177, 309–317. 10.1007/s11046-014-9753-5 24804977

[B18] Jenkinson H. F. Douglas L. J. (2002). Interactions between candida species and bacteria in mixed infections, in Polymicrobial Diseases, eds Brogden K. A. Guthmiller J. M. (Washington, DC: ASM Press), 18–19. 21735561

[B19] Kanafani Z. A. Perfect J. R. (2008). Resistance to antifungal agents: mechanisms and clinical impact. Clin. Infect. Dis. 46, 120–128. 10.1086/524071 18171227

[B20] Katiraee F. Teifoori F. Soltani M. (2015). Emergence of azoles resistance candida species in Iranian AIDS defined patients with oropharyngeal candidiasis. Curr. Med. Mycol. 1, 11–16. 10.18869/acadpub.cmm.1.3.11 28680991 PMC5490324

[B21] Katragkou A. Tsikopoulou F. Roilides E. Zaoutis T. E. (2012). Posaconazole: when and how? The clinician's view. Mycoses 55, 110–122. 10.1111/j.1439-0507.2011.02061.x 21762211

[B22] Kaur R. Dhakad M. S. Goyal R. Bhalla P. Dewan R. (2016). Spectrum of opportunistic fungal infections in HIV/AIDS patients in tertiary care hospital in India. Can. J. Infect. Dis. Med. Microbiol. 2016:2373424. 10.1155/2016/2373424 27413381 PMC4931070

[B23] Kigen G. Kimaiyo S. Nyandiko W. Faragher B. Sang E. Jakait B.. (2011). Prevalence of potential drug-drug interactions involving antiretroviral drugs in a large Kenyan cohort. PLoS ONE 6:e16800. 10.1371/journal.pone.0016800 21373194 PMC3044141

[B24] Kirti Y. K. Yashveer J. K. Poorey V. K. (2015). Changing trends of HIV/AIDS in otorhinolaryngology with CD4 + count correlation. Indian J. Otolaryngol. Head Neck Surg. 67, 12–15. 10.1007/s12070-014-0712-8 25621247 PMC4298593

[B25] Konaté A. Barro-Kiki P. C. M. Kassi K. F. Angora K. E. Vanga-Bosson H. Djohan V.. (2017). Oropharyngeal candidiasis prevalence among HIV-infected patients at the teaching hospital of Treichville (Abidjan, Côte d'Ivoire). J. Mycol. Med. 27, 549–553. 10.1016/j.mycmed.2017.08.005 28867257

[B26] Kwamin F. Nartey N. O. Codjoe F. S. Newman M. J. (2013). Distribution of candida species among HIV-positive patients with oropharyngeal candidiasis in Accra, Ghana. J. Infect. Dev. Ctries. 7, 41–45. 10.3855/jidc.2442 23324819

[B27] Li X. Lei L. Tan D. Jiang L. Zeng X. Dan H.. (2012). Oropharyngeal candida colonization in human immunodeficiency virus infected patients. APMIS 121, 375–402. 10.1111/apm.12006 23030258

[B28] Liu J. Y. Shi C. Wang Y. Li W. J. Zhao Y. Xiang M. J. (2015). Mechanisms of azole resistance in *Candida albicans* clinical isolates from Shanghai, China. Res. Microbiol. 166, 153–161. 10.1016/j.resmic.2015.02.009 25748216

[B29] Lortholary O. Petrikkos G. Akova M. Arendrup M. C. Arikan-Akdagli S. Bassetti M. (2012). ESCMID guideline for the diagnosis and management of candida diseases 2012: patients with HIV infection or AIDS. Clin. Microbiol. Infect. 18, 68–77. 10.1111/1469-0691.12042 23137138

[B30] Maurya V. Srivastava A. Mishra J. Gaind R. Marak R. S. K. Tripathi A. K.. (2013). Oropharyngeal candidiasis and candida colonization in HIV positive patients in Northern India. J. Infect. Dev. Ctries. 7, 608–613. 10.3855/jidc.2801 23949296

[B31] Mehra T. Köberle M. Braunsdorf C. Mailänder-Sanchez D. Borelli C. Schaller M. (2012). Alternative approaches to antifungal therapies. Exp. Dermatol. 21, 778–782. 10.1111/exd.12004 23078400 PMC3481189

[B32] Menezes Rde P. Borges A. S. Araujo L. B. Pedroso R. S. Röder D. V. (2015). Related factors for colonization by candida species in the oral cavity of HIV-infected individuals. Rev. Inst. Med. Trop. Sao Paulo 57, 413–419. 10.1590/S0036-46652015000500008 26603229 PMC4660451

[B33] Morace G. Perdoni F. Borghi E. (2014). Antifungal drug resistance in candida species. J. Glob. Antimicrob. Resist. 2, 254–259. 10.1016/j.jgar.2014.09.002 27873684

[B34] Mulu A. Kassu A. Anagaw B. Moges B. Gelaw A. Alemayehu M.. (2013). Frequent detection of ‘azole’ resistant candida species among late presenting AIDS patients in Northwest Ethiopia. BMC Infect. Dis. 13:82. 10.1186/1471-2334-13-82 23398783 PMC3577436

[B35] Nadagir S. D. Chunchanur S. K. Halesh L. H. Yasmeen K. Chandrasekhar M. R. Patil B. S. (2008). Significance of isolation and drug susceptibility testing of non-*Candida albicans* species causing oropharyngeal candidiasis in HIV patients. Southeast Asian J. Trop. Med. Public Health 39, 492–495. Available online at: https://pdfs.semanticscholar.org/5f83/2d6da119abe25d43465359f9aae343199097.pdf 18564689

[B36] Nett J. E. Marchillo K. Spiegel C. A. Andes D. R. (2010). Development and validation of an *in vivo Candida albicans* biofilm denture model. Infect. Immun. 78, 3650–3659. 10.1128/IAI.00480-10 20605982 PMC2937450

[B37] Patel P. K. Erlandsen J. E. Kirkpatrick W. R. Berg D. K. Westbrook S. D. Louden C.. (2012). The changing epidemiology of oropharyngeal candidiasis in patients with HIV/AIDS in the era of antiretroviral therapy. AIDS Res. Treat. 2012:262471. 10.1155/2012/262471 22970352 PMC3434376

[B38] Patil S. Rao R. S. Majumdar B. Anil S. (2015). Clinical appearance of oral candida infection and therapeutic strategies. Front. Microbiol. 6:1391. 10.3389/fmicb.2015.01391 26733948 PMC4681845

[B39] Patton L. L. Bonito A. J. Shugars D. A. (2001). A systematic review of the effectiveness of antifungal drugs for the prevention and treatment of oropharyngeal candidiasis in HIV-positive patients. Oral Surg. Oral Med. Oral Pathol. Oral Radiol. Endod. 92, 170–179. 10.1067/moe.2001.116600 11505264

[B40] Pfaller M. A. (2012). Antifungal drug resistance: mechanisms, epidemiology, and consequences for treatment. Am. J. Med. 125, S3–S13. 10.1016/j.amjmed.2011.11.001 22196207

[B41] Ramírez-Amador V. A. Espinosa E. González-Ramírez I. Anaya-Saavedra G. Ormsby C. E. Reyes-Terán G. (2009). Identification of oral candidosis, hairy leukoplakia and recurrent oral ulcers as distinct cases of immune reconstitution inflammatory syndrome. Int. J. STD AIDS 20, 259–261. 10.1258/ijsa.2008.008351 19304971

[B42] Repentigny L. Lewandowski D. Jolicoeur P. (2004). Immunopathogenesis of oropharyngeal candidiasis in human immunodeficiency virus infection. Clin. Microbiol. Rev. 17, 729–759. 10.1128/CMR.17.4.729-759.2004 15489345 PMC523562

[B43] Rosana Y. Lestari D. C. Yasmon A. (2015). Overexpression and mutation as a genetic mechanism of fluconazole resistance in *Candida albicans* isolated from human immunodeficiency virus patients in Indonesia. J. Med. Microbiol. 64, 1046–1052. 10.1099/jmm.0.000123 26297039

[B44] Rossoni R. D. Barbosa J. O. Vilela S. F. dos Santos J. D. de Barros P. P. Prata M. C.. (2015). Competitive interactions between *C. albicans, C. glabrata* and *C. krusei* during biofilm formation and development of experimental candidiasis. PLoS ONE 10:e0131700. 10.1371/journal.pone.0131700 26146832 PMC4493022

[B45] Salari S. Khosravi A. R. Mousavi S. A. A. Nikbakht-Brojeni G. H. (2016). Mechanisms of resistance to fluconazole in *Candida albicans* clinical isolates from Iranian HIV-infected patients with oropharyngeal candidiasis. J. Mycol. Med. 26, 35–41. 10.1016/j.mycmed.2015.10.007 26627124

[B46] Sanglard D. (2016). Emerging threats in antifungal-resistant fungal pathogens. Front. Med. 3:11. 10.3389/fmed.2016.00011 27014694 PMC4791369

[B47] Sanguinetti M. Posteraro B. Lass-Flörl C. (2015). Antifungal drug resistance among candida species: mechanisms and clinical impact. Mycoses 58, 2–13. 10.1111/myc.12330 26033251

[B48] Sardi Jde C. Pitangui Nde S. Rodríguez-Arellanes G. Taylor M. L. Fusco-Almeida A. M. Mendes-Giannini M. J. S. (2014). Highlights in pathogenic fungal biofilms. Rev. Iberoam. Micol. 31, 22–29. 10.1016/j.riam.2013.09.014 24252828

[B49] Sharifzadeh A. Khosravi A. R. Shokri H. Jamnani F. A. Hajiabdolbaghi M. Tamami I. A. (2013). Oral microflora and their relation to risk factors in HIV + patients with oropharyngeal candidiasis. J. Mycol. Med. 23, 105–112. 10.1016/j.mycmed.2013.02.001 23721997

[B50] Sharifzadeh A. Khosravi A. R. Shokri H. Sharafi G. (2015). Antifungal effect of *Trachyspermum ammi* against susceptible and fluconazole-resistant strains of *Candida albicans*. J. Mycol. Med. 25, 143–150. 10.1016/j.mycmed.2015.03.008 25982599

[B51] Swart A. Harris V. (2005). Drug interactions with tuberculosis therapy: main topic. Community Med. Educ. 23, 56–60. Available online at: http://www.cmej.org.za/index.php/cmej/article/view/608

[B52] Tamí-Maury I. Willig J. Vermund S. Jolly P. Aban I. Hill J.. (2011). Contemporary profile of oral manifestations of HIV/AIDS and associated risk factors in a Southeastern US clinic. J. Public Health Dent. 71, 257–264. 10.1111/j.1752-7325.2011.00256.x 22320283

[B53] Tati S. Davidow P. McCall A. Hwang-Wong E. Rojas I. G. Cormack B.. (2016). *Candida glabrata* binding to *Candida albicans* hyphae enables its development in oropharyngeal candidiasis. PLoS Pathog. 12:e1005522. 10.1371/journal.ppat.1005522 27029023 PMC4814137

[B54] Terças A. L. G. Marques S. G. Moffa E. B. Alves M. B. de Azevedo C. M. P. S. Siqueira W. L.. (2017). Antifungal drug susceptibility of candida species isolated from HIV-positive patients recruited at a public hospital in São Luís, Maranhão, Brazil. Front. Microbiol. 8:298. 10.3389/fmicb.2017.00298 28303122 PMC5332371

[B55] Thein Z. M. Seneviratne C. J. Samaranayake Y. H. Samaranayake L. P. (2009). Community lifestyle of Candida in mixed biofilms: a mini review. Mycoses 52, 467–475. 10.1111/j.1439-0507.2009.01719.x 19486299

[B56] Thompson G. R. III. Patel P. K. Kirkpatrick W. R. Westbrook S. D. Berg D. Erlandsen J.. (2010). Oropharyngeal candidiasis in the era of antiretroviral therapy. Oral Surg. Oral Med. Oral Pathol. Oral Radiol. Endod. 109, 488–495. 10.1016/j.tripleo.2009.11.026 20156694 PMC2843789

[B57] Vandeputte P. Ferrari S. Coste A. T. (2012). Antifungal resistance and new strategies to control fungal infections. Int. J. Microbiol. 2012:713687. 10.1155/2012/713687 22187560 PMC3236459

[B58] Vazquez J. (2010). Optimal management of oropharyngeal and esophageal candidiasis in patients living with HIV infection. HIV AIDS 2, 89–101. 10.2147/HIV.S6660 22096388 PMC3218701

[B59] Vazquez J. A. (2000). Therapeutic options for the management of oropharyngeal and esophageal candidiasis in HIV/AIDS Patients. HIV Clin. Trials 1, 47–59. 10.1310/T7A7-1E63-2KA0-JKWD 11590489

[B60] Walker N. F. Scriven J. Meintjes G. Wilkinson R. J. (2015). Immune reconstitution inflammatory syndrome in HIV-infected patients. HIV AIDS 7, 49–64. 10.2147/HIV.S42328 25709503 PMC4334287

[B61] Zhang J. Silao F. G. S. Bigol U. G. Bungay A. A. C. Nicolas M. G. Heitman J.. (2012). Calcineurin is required for pseudohyphal growth, virulence, and drug resistance in *Candida lusitaniae*. PLoS ONE 7:e44192. 10.1371/journal.pone.0044192 22952924 PMC3432075

[B62] Zheng S. Chang W. Zhang M. Shi H. Lou H. (2018). Chiloscyphenol A derived from Chinese liverworts exerts fungicidal action by eliciting both mitochondrial dysfunction and plasma membrane destruction. Sci. Rep. 8:326. 10.1038/s41598-017-18717-9 29321629 PMC5762906

